# The Influence of Endogenous and Exogenous Spatial Attention on Decision Confidence

**DOI:** 10.1038/s41598-017-06715-w

**Published:** 2017-07-25

**Authors:** Phillipp Kurtz, Katharine A. Shapcott, Jochen Kaiser, Joscha T. Schmiedt, Michael C. Schmid

**Affiliations:** 1 Ernst Strüngmann Institute (ESI) for Neuroscience in cooperation with Max Planck Society, Frankfurt, Germany; 2 Institute of Medical Psychology, Goethe University, Frankfurt am Main, Germany; 3 Institute of Neuroscience, Newcastle University, Newcastle upon Tyne, UK

## Abstract

Spatial attention allows us to make more accurate decisions about events in our environment. Decision confidence is thought to be intimately linked to the decision making process as confidence ratings are tightly coupled to decision accuracy. While both spatial attention and decision confidence have been subjected to extensive research, surprisingly little is known about the interaction between these two processes. Since attention increases performance it might be expected that confidence would also increase. However, two studies investigating the effects of endogenous attention on decision confidence found contradictory results. Here we investigated the effects of two distinct forms of spatial attention on decision confidence; endogenous attention and exogenous attention. We used an orientation-matching task, comparing the two attention conditions (endogenous and exogenous) to a control condition without directed attention. Participants performed better under both attention conditions than in the control condition. Higher confidence ratings than the control condition were found under endogenous attention but not under exogenous attention. This finding suggests that while attention can increase confidence ratings, it must be voluntarily deployed for this increase to take place. We discuss possible implications of this relative overconfidence found only during endogenous attention with respect to the theoretical background of decision confidence.

## Introduction

Spatial attention is a fundamental aspect of everyday life that helps us carry out efficient perceptual decisions. The literature differentiates between two forms of spatial attention: endogenous (or top-down) attention is voluntary deployed and sustained^1^, exogenous (or bottom-up) attention however, occurs reflexively^2^. ‘Reflexive’ and ‘voluntary’ in this context refer to the finding that peripheral cues (i.e. placed near or directly at the experimental target stimulus) that are used to guide exogenous attention cannot be ignored or interrupted voluntarily^2, 3^, whereas central cues (i.e. placed away from the target, often around the fixation point) for endogenous attention rely on the validity of the cue and the willingness or cognitive control of the subjects to deploy their attention^3^. Additionally, time courses are different between the two forms of attention: Exogenous attention is very rapidly deployed. It takes only 90–120 ms until an attention effect with a peripheral cue can be detected, yet benefits only last until 300 ms following cue onset^1, 4, 5^. Endogenous attention on the other hand is engaged only 300–500 ms after onset of a central cue^1, 4, 5^ but can be kept at one location for at least 1200 ms^1^. Therefore, due to the different phenotypes of these two forms of spatial attention they are believed to arise from distinct neuronal mechanisms^6, 7^ and might differently affect perceptual decision making. Previous research established that endogenous attention can increase perceptual decision accuracy^8–11^. Whether exogenous attention has a similar effect is not well understood. A primary goal of this study was therefore to directly compare the effects on endogenous vs exogenous spatial attention on perceptual decision accuracy.

A second goal was to understand the benefits of attention on decisions beyond accuracy, namely on decision confidence. Decision confidence describes the probability that a decision is correct or accurate as estimated by the subjects themselves given the evidence available^12^. People can intuitively report confidence with numerical ratings^13^ or on a continuous scale^14, 15^. Recent neurophysiological studies have argued that this is so easy because an evaluation of the quality of the evidence is inherent to every decision process^16, 17^. Several models of decision confidence therefore assume that the sensory evidence (i.e. the information about the stimulus that is available to the subject) is the major input to the computation of confidence ratings^14, 16, 18–21^. In simplified terms these models compute a decision variable based on accumulated sensory evidence which determines the choice the subject makes in the task. Decision confidence is then derived from the strength of the sensory evidence for the respective decision. Indeed, confidence is coupled to the objective quality of a decision (performance)^22, 23^, but it can also be modulated by a number of contextual factors. For example task type^24^, task instruction^23^, feedback^25^, decision time^26^ and individual differences^27^ all influence decision confidence. Recent psychophysical and neurophysiological studies have even suggested a separate process for the calculation of confidence^13, 28, 29^.

Might spatial attention influence perceptual confidence? If confidence and performance are a result of the very same brain process, one might expect a positive effect of attention on both. If however, confidence and performance arise from separate processes, attention may affect both differently. To the authors’ knowledge only two studies so far have investigated the influence of spatial attention on perceptual confidence. A study by Wilimzig *et al*.^30^ reported that spatial attention has no influence on decision confidence. In contrast, Zizlsperger *et al*.^29^ found that both spatial and feature-based attention have larger effects on confidence than on performance, and may even cause over-confidence. They argued therefore that decisions and decision confidence are likely to arise from at least partially separate brain processes, which are differently influenced by endogenous attention.

In this study we provide supporting evidence for the findings of Zizlsperger *et al*. on the effects of endogenous attention. In addition, we show that this finding cannot be extended to exogenous attention. While both endogenous and exogenous attention enhanced performance, only endogenous attention increased confidence ratings. We show that this increase could not be explained solely by enhanced performance, which indicates relative overconfidence resulting from endogenous attention.

Therefore, our results demonstrate that it is only the voluntary (endogenous) form of attention that affects both performance and confidence while the reflexive (exogenous) form of attention affects performance but not confidence.

## Results

**Figure 1:**
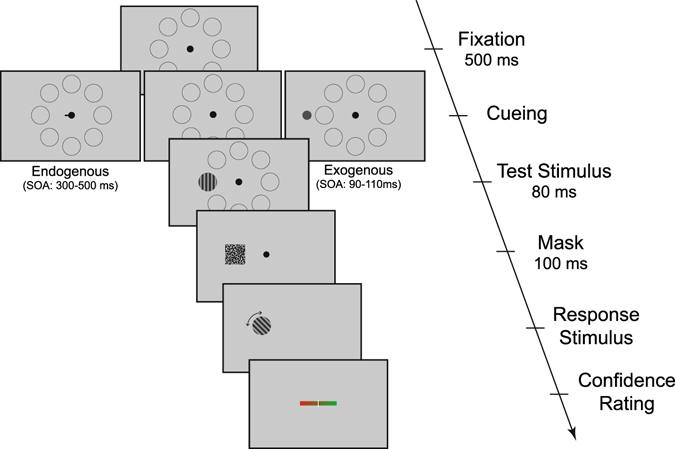
Orientation matching task. Participants attempted to reproduce the orientation of a grating test stimulus using arrow keys to turn the response stimulus. Afterwards they reported the confidence in their decision on a continuous scale, again using arrow keys. During the fixation and cueing period eight grey circles indicated the possible locations at which the grating could appear. The average trial time was the same across the three conditions and the only difference in the trial sequence between conditions was during the cueing period. In the endogenous condition a foveally presented “Posner” line pointed for 300–500 ms to the location where the stimulus would appear. In the exogenous condition a small grey dot was briefly (16 ms) flashed immediately next to the location of the target. Stimulus onset asynchrony (SOA) between cue and target onset was 90–110 ms in the exogenous condition. Both cues were 100% valid.

28 participants (23 were analyzed, see Methods) performed an orientation matching task with sinusoidal gratings under three different conditions: endogenous attention with central cueing, exogenous attention with peripheral cueing and a control condition without cueing. Every participant performed 100 trials per condition. At the end of each trial, participants gave a confidence rating about their performance in the respective trial (Fig. 1).

**Figure 2:**
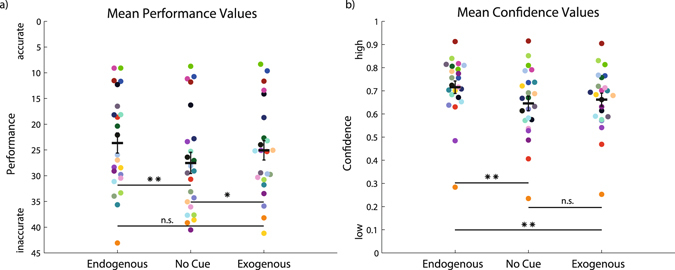
Performance and confidence compared across attention conditions. (**a**) Distribution of subject-averaged performance data. Every dot represents the mean performance of one participant in the respective condition. Every color corresponds to one participant showing how the individual participant contributed to the observed result. Both endogenous (left) and exogenous (right) attention conditions have greater means (grand average) than the no-cue condition (center). (**b**) Distribution of subject-averaged confidence data. Note the similarity of the exogenous and no-cue distributions. Means are indicated in black. Levels of significance were computed using post-hoc comparisons following a one-way repeated-measures ANOVA on mean values of participants. Asterisks denote significant results of the post-hoc comparison; **p < 0.01, *p < 0.05, n.s.: not significant (p > 0.05). Error bars are standard error of the mean.

To assess the influence of attention, we initially compared whether performance and confidence differed between a condition without cue and endogenous and exogenous cueing. Figure 2a shows mean values for performance in every subject. Performance was more accurate when attention was endogenously or exogenously cued compared with the no-cue condition. To quantify this effect and summarize it across all subjects, a one-way repeated-measures ANOVA confirmed a significant effect of attention condition on performance (F (df = 2, 44) = 7.48, p = 0.002). Compared to the no-cue condition (27.5° ± 9.95°), performance was significantly higher under endogenous attention (23.6° ± 9.41°) (t (df = 22) = 3.5, p = 0.006). Similarly, performance was significantly better under exogenous attention (25.08° ± 9.03°) compared to the no cue condition (t (df = 22) = 2.87, p = 0.027). Although there was a small trend for better performance under exogenous compared to endogenous attention, the difference was not statistically significant (t (df = 22) = 1.35, p = 0.57).

For decision confidence a slightly different pattern was observed. The distribution for endogenous attention shows that mean confidence ratings were higher than in the other two conditions (Fig. 2b). As the distributions of confidence values were not normally distributed, we used a non-parametric Friedman test to statistically compare the effect of attention condition on confidence ratings. This gave a Chi-square value of 12.9 (df = 2, 44), which was significant (p = 0.002). Post-hoc comparison with Wilcoxon signed rank tests indicated a difference between median confidence in the no-cue condition (median = 0.67, mean = 0.65 ± standard deviation = 0.15) and in the endogenous attention condition (0.72, 0.72 ± 0.13) (Z = −3.25, p = 0.003). In addition, mean confidence in the endogenous attention was higher than in the exogenous attention condition (0.68, 0.66 ± 0.13) (Z = 3.41, p = 0.002). However, there was no difference between exogenous attention and no-cue conditions (Z = −0.91, p = 1). Thus, while both attention conditions enhanced performance, only endogenous cueing resulted in increased confidence ratings.

**Figure 3:**
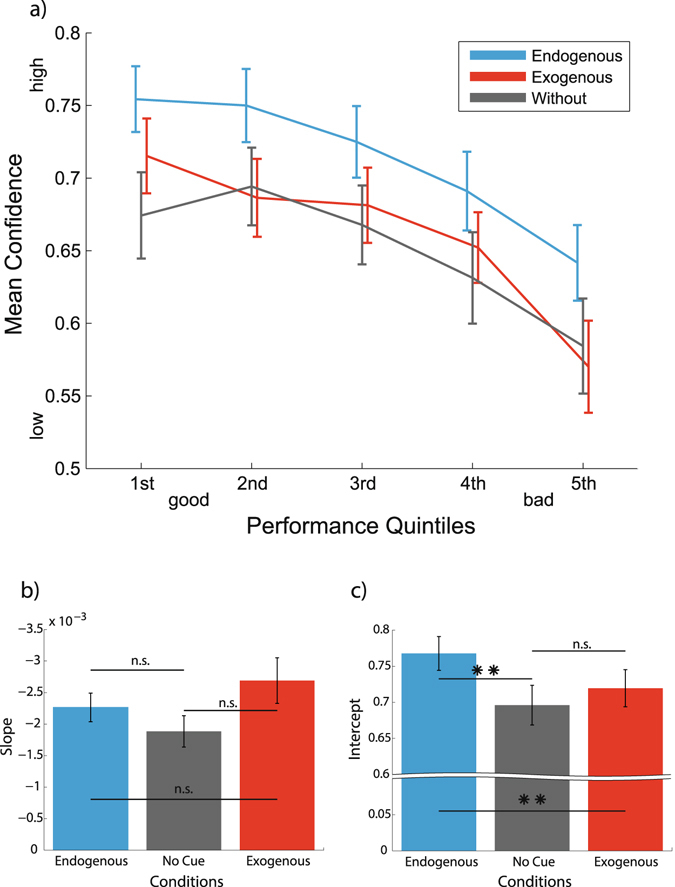
Relationship between confidence and performance. (**a**) Mean confidence is higher in the endogenous attention condition for a wide range of performances. For this plot we binned single subject data into quintiles based on performance. Shown is the grand average for confidence in the respective performance quintile. Error bars are standard error of the mean. (**b–c**) A linear model was fit to the data for every participant for every attentional condition. Shown are the mean values for the slope (**b**) and the intercept (**c**) of the linear fit for every condition respectively. Asterisks denote significant results of the post-hoc comparison; **p < 0.01, *p < 0.05, n.s.: not significant (p > 0.05). Error bars are standard error of the mean.

We then investigated whether attention changed the relationship between performance and confidence. To accommodate for the different distributions underlying the confidence vs performance measures, we binned single subject data into quintiles based on performance. In every participant the mean confidence rating for all trials in the respective performance quintile was calculated for every condition (Fig. 3a). A two-way (3 attention conditions x 5 performance bins) repeated measures ANOVA yielded main effects of attention (F (df = 2,44) = 11.75, p < 0.001) and performance (F (df = 4,88) = 45.25, p < 0.001), while there was no statistically significant interaction between both factors (F (df = 8, 176) = 1.73, p = 0.09). Hence, endogenous attention led to higher confidence ratings irrespective of the performance level. This showed that the increase in confidence with endogenous attention was not just a faithful reflection of enhanced performance, but rather that trials with equal performance showed higher confidence with endogenous attention than in the control condition. We call this a relative over-confidence. Exogenous attention led to higher performance but not to higher confidence ratings. Relative overconfidence was therefore found selectively for endogenous attention.

To further objectify the impression in Fig. 3a that endogenous attention led to an upwards shift of the performance-confidence relationship, a linear model was fit to the data for every participant for every attentional condition. On the results of the fitting parameters a repeated-measures ANOVA was conducted separately for slope (see Fig. 3b) and intercept (see Fig. 3c) of the fitted relationship. This showed that while there was no significant effect of condition on the slope of the linear fit (F (df = 2, 44) = 1.98, p = 0.1502), the intercept differed significantly between conditions (F (df = 2, 44) = 14.4, p < 0.0001). Multiple comparisons revealed that the endogenous condition had a significantly higher intercept than both no-cue (t (22) = 4.57, p < 0.001) and exogenous conditions (t (22) = 3.5, p = 0.006). Again there was no difference between no-cue and exogenous condition (t (22) = −2.14, p = 0.13). We therefore concluded that endogenous attention led to an upward shift of the performance-confidence relationship leaving the slope of the relation intact. In contrast, exogenous attention left this relationship unaffected. This analysis showed that the relative overconfidence we found for endogenous attention was not just an effect of binning the data. More elaborate statistical analyses techniques examining interactions or using performance as covariate (see Supplementary Analysis S1) confirmed these results.

Finally we wanted to confirm that the correlation between performance and confidence was also not just an effect of artificial binning. Therefore, we calculated Spearman’s rho for every participant and ran a one-sample t-test across participants. This analysis confirmed that in every condition the correlation was statistically significant (no-cue: mean = −0.18 ± standard deviation = 0.12, t (22) = −7.39, p < 0.001, endogenous: −0.22 ± 0.11, t (22) = −9.51, p < 0.001, exogenous: −0.23 ± 0.12, t (22) = −9.33, p < 0.001). However, using a one-way repeated measures ANOVA no statistical difference between conditions could be detected (F (df = 2, 44) = 1.58, p = 0.22). This analysis confirmed there was a correlation between performance and confidence and that this correlation was comparable across conditions.

One potential caveat of these results might be that confidence was heavily clustered in the high confidence range. There was a tendency in some of the subjects to report the highest possible confidence. However, even after exclusion of all trials in which the highest possible confidence was reported, neither the effect of attention condition on performance nor on confidence changed qualitatively. Results of the repeated-measures ANOVA and following multiple comparisons are given in Supplementary Tables S2–S5.

Another possibility was that participants might have performed or rated confidence differently depending on the difference between starting orientation of the response grating and the recalled stimulus. This might have been because it felt less difficult or was less difficult to match the orientations if this difference was small. If the orientation difference did influence performance or confidence, then we should have found that these measures were correlated to it. Therefore, we calculated this correlation using Spearman’s rho for the 15 subjects for which the difference in orientation was recorded. We then performed a one-sample t-test across participants for the correlation with both performance and confidence. This yielded no significant result, neither for performance (t (df = 14) = −0.16, p = 0.88) nor for confidence (t (df = 14) = 1.12, p = 0.28). A mediating effect of the starting orientation is therefore unlikely.

A final confound that we examined was whether systematic differences in gaze position towards the cued location and therefore differential sensory input might account for the observed effects. Details of this analysis are provided in supplementary material (see Supplementary Figure S6). Gaze positions were not significantly directed towards the cued location in either of the attention conditions and therefore could not explain the observed differences between the attention conditions.

## Discussion

We compared the effects of endogenous and exogenous spatial attention on performance and confidence during a perceptual decision task. While both forms of attention led to an increase in performance, only endogenous attention also led to higher confidence ratings. Additionally we found that endogenous attention went, in some cases, beyond an accurate reflection of the enhanced performance and led to relative overconfidence. We found higher confidence with endogenous attention compared to the other conditions even when performance was poor. We showed that this effect could not be observed with exogenous attention, which did not change the relationship between performance and confidence.

Two previous studies examined the effects of endogenous spatial attention on decision performance and confidence and reported discrepant findings. Our work supports the findings by Zizlsperger *et al*.^29^ that endogenous attention increases decision confidence more than performance. Wilimzig *et al*.^30^ on the other hand observed no effect of attention on confidence. As Zizlsperger *et al*. already pointed out this might be because Wilimzig *et al*. instructed participants to answer “as fast and as accurate as possible” because this might have caused subjects to rate their confidence before the computation of it was completed. Furthermore, in Wilimzig *et al*.’s task performance increased with attention but confidence did not, perhaps due to a ceiling effect. If a task is very easy, participants still sometimes report low confidence. This has been attributed to “a general form of underconfidence”^22, 31^ as little effort is required and successes can be attributed to the task design. This might have been the case in the attended condition in Wilimzig *et al*.’s task, since in their yes/no paradigm performance could never be below 50% correct. Zizlsperger *et al*. used 4 possible answers and we, at least in theory, provided 180, which substantially increased overall uncertainty and difficulty of the decision and therefore should have prevented a ceiling effect.

The dissociation between the effects of the two forms of attention adds supporting evidence for the hypothesis that endogenous and exogenous attention are separate processes^4, 32^. The neural networks for orienting spatial attention have not yet been identified unambiguously but it has been proposed that at least two different networks exist that might correspond to endogenous and exogenous attention^7^ (but see refs 33, 34). The neural network for decision confidence^16, 20^ overlaps at least in part with the network linked to endogenous attention in the intraparietal sulcus and in the pulvinar nucleus of the visual thalamus^35, 36^. How attention and decision confidence are integrated at the neuronal level is still largely unknown. The results of our experiments indicate that under the tested conditions, endogenous, but not exogenous, attention will influence decision confidence. Neurophysiological recordings could test for possible neural networks which are responsible for the effects of the different forms of attention.

Previous studies on decision confidence have focused on whether confidence is computed at the time point of the decision (decisional locus model, see for example ref. 18) or whether it takes information into account that was perceived after the decision (post-decisional locus model, see for example ref. 37) (for a review see ref. 38). However, these models assume that confidence represents the probability of the decision being correct and can therefore in some way be read out from the sensory evidence on which the decision is based^14, 16, 17, 19–21^. A relatively new theory proposes that confidence could be obtained in a process that is separated from the decision and can therefore selectively be manipulated (i.e. without manipulating the sensory evidence)^13, 28, 29^. Whether the objective (performance) and subjective evaluation of a decision (confidence) arise from the same or from different processes remains an open question. Our findings add supporting evidence in favor of a separation between these processes.

In theory both forms of attention could in some way affect the evidence accumulation process and consequently the decision variable, which determines the decision in a positive manner. This is reflected in the increased performance in both attention conditions. If confidence was obtained mainly depending on the strength of the sensory evidence for the respective decision - as suggested by many models^14, 16, 18–21^ - one would expect that it would be affected by attention in the same way as the quality of the decision itself (performance). In our study however, confidence was only affected by one form of attention. During exogenous attention, confidence faithfully reflected the quality of a decision. For endogenous attention we found selective relative overconfidence relative to performance. This dissociation can only be explained when we assume a second, separate process for obtaining confidence ratings. While attention can improve the first process (performance), it seems to have an even stronger effect on the second process (confidence). It is the voluntary, or cognitively controlled, form of attention that affects this process while the reflexive, or involuntary, form of attention leaves it unchanged, suggesting that confidence might ultimately arise from higher cognitive processes. Another finding that might potentially link confidence to higher cognitive processes is that participants are more likely to be overconfident in higher cognitive tasks than in perceptual tasks^24^ (but see ref. 39). Additionally neural correlates of confidence have been found in prefrontal areas^40^, thought to be important for cognitive control^41^. Taken together, these considerations set confidence apart from the externally triggered perceptual decision process itself and link it to metacognitive processes, i.e. monitoring mechanisms over perception or memory using high level control^42^.

One limitation to this study is that there is no possibility to validate post-hoc that the experimental manipulations really resulted in the deployment of different forms of attention. This claim relies on the well documented differences in the time courses between exogenous attention and endogenous attention^1, 4, 5, 43, 44^. Another possible caveat is that attention might not affect confidence as such but simply the reporting process of this internal confidence value. For example, participants might have only reported “certain” rather than truly felt this way during their confidence rating, simply because they thought they should be more certain under this task condition. It is also possible that in the exogenous attention condition participants felt rushed by the short SOA or were surprised by the cue and therefore reduced their confidence ratings accordingly (However, an effect of SOA could be ruled out at least within conditions (see Supplementary Analysis S7)). In our experiment these factors cannot be ruled out completely and our results should therefore be further validated in combination with a greater range of psychological methods assessing metacognitive processes^45^.

## Conclusions

Reflexive attention to external information does not affect the assessment of a decision in a metacognitive process even if it enhances the objective quality of the decision. Deciding voluntarily to do so on the other hand may lead us to overestimate the quality of our decisions. Thus, higher level cognitive processes can influence each other, while they appear to be at least partially decoupled from the basic reflexive processes that help us perceive our environment in an optimal manner.

## Methods

### Subjects

28 healthy participants performed the task (12 female, median age: 25 years). All participants had normal or corrected to normal vision. Written informed consent was obtained from all subjects. All methods were carried out in accordance with relevant guidelines and regulations and approved by the ethics committee of the Goethe University Medical Faculty. Participants 1–10 received small gifts for their participation, participants 11–28 were paid € 15. Apart from 4 participants from the author’s lab (including the first author), all participants were naïve to the task. Performing the task including training trials, giving informed consent, task instructions (See Supplementary Information S8) and pauses took between one and one and a half hours.

Five participants (8, 9, 13, 21, and 26) performed at chance level and were therefore excluded from analysis. We determined participants at chance level by using their median accuracy, which from random guesses between 0 and 90 would be around 45°, and excluded all participants with a median accuracy larger than 40°. However, excluding these participants did not qualitatively change the observed effect.

Task settings were slightly changed from subject 17 onwards (see below) because of feedback from participants without experience in psychophysical experiments. Comparing the descriptive statistics between the first and second group of participants we found that performance was poorer in the second group in all conditions (endogenous: mean = 18.41° ± standard deviation = 8.36 vs. 30.44° ± 5.67°; exogenous: 21.95° ± 10.49° vs. 29.15° ± 4.49°; no-cue: 22.77° ± 9.97° vs. 33.71° ± 5.84°). This was reflected also by lower confidence ratings (endogenous: 0.74 ± 0.083 vs. 0.68 ± 0.172; exogenous: 0.68 ± 0.116 vs. 0.64 ± 0.159; no-cue: 0.68 ± 0.128 vs. 0.6 ± 0.173). We attribute this to the fact that the second group included only participants without experience in psychophysical experiments. The main differences between conditions, however, were very similar for both groups. Therefore, all participants were treated as one population.

### Task Design

The experiment was performed in a quiet, dimly lit room. Participants placed their head in a headrest ensuring a constant viewing distance of approximately 60 cm. Stimuli were presented on a Samsung SyncMaster 2233RZ monitor with a resolution of 1680 by 1050 pixels and a refresh rate of 120 Hz^46^. Presentation was controlled by a Dell Computer with an Intel Xeon W3503 processor (2.4 GHz) and a NVIDIA Quadro 2000D graphics card. The operating system was a 64-bit Windows 7 Professional. The experimental procedure was programmed using the Psychtoolbox version 3.0.12^47^ for Matlab version R2014b (Mathworks Inc. TM).

The orientation matching procedure shown in Fig. 1 was based on a paradigm used by Whitney *et al*.^48^. All stimuli were presented on a grey background. Participants had to fixate a small fixation point with a diameter of 0.2 visual degrees to start a trial. Eight light grey circles indicated possible stimulus locations at 5 degrees eccentricity. This presentation stayed on for 500 milliseconds (ms) after fixation was acquired and was the same in all trials.

Three different conditions were tested: endogenous versus exogenous cueing and a no-cue condition. Conditions were pseudo-randomly drawn on a trial-by-trial basis so that every condition was tested in exactly one third of the trials.

In the no-cue condition the initial presentation stayed on the screen for an additional 300–700 ms (from participant 17 onwards for 350–750 ms) after the initial 500 ms fixation period until the stimulus was presented 800–1200 ms after trial onset. This condition served as a control condition to assess decision performance and confidence in the absence of cued attention.

In the endogenous attention condition a 1 visual degree long black line pointing from the central fixation spot towards one of the locations appeared within 200 ms after the end of the fixation period and stayed on for a duration of 300–500 ms (from participant 17 onwards for 350–550 ms) instructing the subjects to shift their attention covertly (without detectable eye movements) to the indicated location. Offset of the cue and onset of the target happened simultaneously in this condition.

In the exogenous attention condition after an additional 200–600 ms (from participant 17 onwards: 250–650 ms) of the initial display, a small dark grey dot (0.5 visual degrees diameter) was flashed for 16 ms next to the location where the stimulus would later appear reflexively drawing the subjects’ attention to that location. In this condition the time between cue onset and target onset was 90–110 ms. These stimulation times were selected to accommodate the known time courses of endogenous and exogenous attention^1, 4, 5, 43, 44^.

The stimulus was a circular (2 visual degrees diameter) sinusoidal grating. Michelson contrast was between 0.05 and 0.5 and was set according to performance in a staircase procedure during a prior psychophysical threshold measurement (see below). The spatial frequency of the stimulus and response grating was 6 cycles per degree, which yielded robust effects for exogenous attention in a similar task^49^. The grating’s orientation was randomly drawn on every trial. The target stimulus was presented for 80 ms followed by a random white noise mask that was presented for 100 ms to prevent afterimages that could influence the decision process.

After a 500 ms period with a blank screen, a second randomly oriented response grating was presented. Participants tried to match the orientation of the response grating to the orientation of the stimulus grating by changing the orientation of the response grating in steps of 1° with the arrow (left and right) keys on a keyboard. When satisfied with the orientation of the gratings, participants pressed another key to log in their response. Then participants rated the confidence in their decision on a continuous scale again using the arrow keys to move a slider on the scale. The scale showed the transmission from red on the left side of the scale corresponding to low confidence, to green on the right side of the scale for high confidence. The starting point of the slider was set to the right edge. A value read out from the position of the slider on the scale between 0 for lowest possible confidence to 1 for highest possible confidence was recorded. The scale was divided into 256 possible values corresponding to a span of 256 pixels, giving the impression of a continuous scale. The length of the scale in visual degrees was 6.94.

Participants received an oral instruction either in English or German, which did not contain speed or accuracy statements. Then they performed a training session of 30 to 50 trials using the exogenous cue. These trials were used to determine an individual Michelson contrast for the gratings for each subject with a staircase function. Since the response was given on a continuous scale we used 15° deviation as a correct/incorrect criterion in the staircase procedure. A correct response decreased, and an incorrect response increased the contrast by a step of 0.05. We then used the lowest contrast at which participants were able to perform better than the threshold for the main experiment. In the actual experiment participants performed a total of 300 trials (100 trials per condition).

Additionally we recorded the starting angle of the response grating from participant 11 onwards to check for possible influences on responses. The SOA for endogenous trials was prolonged to 350–550 ms for subject 17–28 because we noticed that participants took longer than expected to allocate attention according to the cue. In trials with exogenous attention cues and in trials without cueing we prolonged the waiting time accordingly to obtain consistent trial lengths.

### Eye-Tracking

The participant’s eye movements and pupil diameter were recorded throughout the whole experiment to ensure fixation with an Eyelink 1000 Version 4.56 system from SR Research Ltd. (Mississauga, Ontario, Canada). Gaze position was used to initiate a trial as soon as it reached the central fixation window. Trials were aborted if gaze position went outside the fixation window before the fixation point was turned off. Eye-movement data was saved from subject 7 onwards. The directionality of the eye position was assessed using a Rayleigh test.

### Statistics

All statistical tests were programmed using the Matlab version 2011a and 2015b and its Statistics and Machine Learning Toolboxes (Mathworks Inc.). The two-way repeated measures ANOVA was performed using the RMAOV2 function^50^.

Performance was calculated as the absolute deviation between the response orientation that participants had logged in and the orientation of the stimulus grating. Confidence was measured as a value between 0 and 1 in 256 steps, read out from final position of the slider on the scale.

To assess the statistical significance of effects across participants, we conducted one-way repeated-measures analyses of variance (ANOVA) for performance and a Friedman test for confidence. When these tests revealed a significant effect (p < 0.05), post-hoc statistical comparison was performed using paired samples t-tests for performance and Wilcoxon signed rank tests for confidence with a Bonferroni procedure correcting for multiple comparisons.

For the analysis of confidence according to performance level single subject data was binned into quintiles and mean confidence values of trials in each condition were calculated for every quintile (Fig. 3). Then a two-way (3 attention conditions x 5 performance levels) repeated measures ANOVA was performed to assess statistical significance.

## Electronic supplementary material

Supplementary Material
