# Supplementary material for: The Influence of Endogenous and Exogenous Spatial Attention on Decision Confidence
Source: Sci Rep. 2017 Jul 25;7:6431. doi: 10.1038/s41598-017-06715-w (PMC5527098; doi:10.1038/s41598-017-06715-w)
Supplement: Supplementary file 1 — Supplementary Material [file 41598_2017_6715_MOESM1_ESM.pdf]

The Influence of Endogenous and Exogenous Spatial Attention on Decision Confidence

Phillipp Kurtz [1], Katharine A. Shapcott [1], Jochen Kaiser [2], Joscha T. Schmiedt[1], Michael C. Schmid [1,3]*

[1] Ernst Strüngmann Institute (ESI) for Neuroscience in cooperation with Max Planck Society, Frankfurt, Germany

[2] Institute of Medical Psychology, Goethe University, Frankfurt am Main, Germany

[3] Institute of Neuroscience, Newcastle University, Newcastle upon Tyne, UK

* Corresponding author

E-Mail: Michael.Schmid@newcastle.ac.uk

Supplementary Information:

S1 Analysis. Non-parametric (permutation-based) Analysis of Covariance (ANCOVA). To get an estimate of the effects of attention on confidence after controlling for performance differences we performed a non-parametric (permutation-based) ANCOVA. After adjusting for performance accuracy, attention continued to have a significant effect on confidence, with $F = 18.75$, $p < 0.001$. The performance adjusted means for confidence under endogenous, exogenous and neutral attention were 0.88, 0.84 and 0.84, respectively. Post-hoc comparison on the adjusted values revealed a significant difference between endogenous and no-cue condition ($t(22) = -3.11$, $p = 0.015$) and between endogenous and exogenous condition ($t(22) = -4$, $p = 0.002$). No significant difference could be detected between exogenous and no-cue condition ($t(22) = 0.088$, $p = 1$). This analysis was done using the Fathom toolbox[52] for Matlab.

SumSq	DF	MeanSq	F	pValue	pValueGG	pValueHF	pValueLB
173.6488	2	86.8244	6.695656	0.00289297	0.00417811	0.00322636	0.0168005
570.5600	44	12.96727	1	0.5	0.5	0.5	0.5

S 2: Repeated-Measures ANOVA for Performance (Highest Confidence Excluded). This table provides the result of the repeated-measures ANOVA for the effect of attention condition on performance after exclusion of all trials where subjects reported full confidence. The effect of attention condition on performance is still significant even after excluding these trials. pValue: p-value for the corresponding F-statistic; pValueGG: p-value with Greenhouse-Geisser adjustment; p-value with Huynh-Feldt adjustment; pValueLB: p-value with Lower bound adjustment.

Conditions_1	Conditions_2	Difference	StdErr	pValue	Lower	Upper
Without	Endogenous	3.8242	1.1782	0.0111	0.7711	6.8773
Without	Exogenous	2.5092	0.8659	0.025	0.2655	4.7528
Endogenous	Exogenous	-1.315	1.1157	0.7534	-4.206	1.576

S 3: Multiple Comparison for Performance (Highest Confidence Excluded). This table provides the results of the multiple comparison for performance after exclusion of trials with full confidence. Still both endogenous and exogenous attention show a significantly higher performance than the no-cue condition. The difference between endogenous and exogenous condition is still not significant. Difference: Estimated difference between the corresponding two marginal mean; StdErr: Standard error of the estimated difference between the corresponding two marginal means; pValue: Bonferroni-corrected p-value; Lower: Lower limit of simultaneous 95% confidence intervals for the true difference; Upper: Upper limit of simultaneous 95% confidence intervals for the true difference.

SumSq	DF	MeanSq	F	pValue	pValueGG	pValueHF	pValueLB
0.0532	2	0.0266	10.4653	0.00019	0.0005	0.00037	0.0038
0.1119	44	0.0025	1	0.5	0.5	0.5	0.5

S 4: Repeated-Measures ANOVA for Confidence (Highest Confidence Excluded). This table provides the results of the repeated-measures ANOVA for the effect of attention condition on confidence after exclusion of all trials where subjects reported full confidence. The effect of attention condition on confidence is still significant even after excluding these trials. pValue: p-value for the corresponding F-statistic; pValueGG: p-value with Greenhouse-Geisser adjustment; p-value with Huynh-Feldt adjustment; pValueLB: p-value with Lower bound adjustment.

Conditions_1	Conditions_2	Difference	StdErr	pValue	Lower	Upper
Without	Endogenous	-0.0652	0.01786	0.0042	-0.11146	-0.01891
Without	Exogenous	-0.0157	0.01234	0.6490	-0.04768	0.01627
Endogenous	Exogenous	0.0495	0.01387	0.0052	0.01354	0.08542

S 5: Multiple Comparison for Performance (Highest Confidence Excluded). This table provides the results of the multiple comparison tests for confidence after exclusion of trials with full confidence. Still endogenous attention resulted in significantly higher confidence than both no-cue condition and exogenous condition. The difference between exogenous and no-cue condition was again not significant. Difference: Estimated difference between the corresponding two marginal mean; StdErr: Standard error of the estimated difference between the corresponding two marginal means; pValue: Bonferroni-corrected p-value; Lower: Lower limit of simultaneous 95% confidence intervals for the true difference; Upper: Upper limit of simultaneous 95% confidence intervals for the true difference.

Endogenous

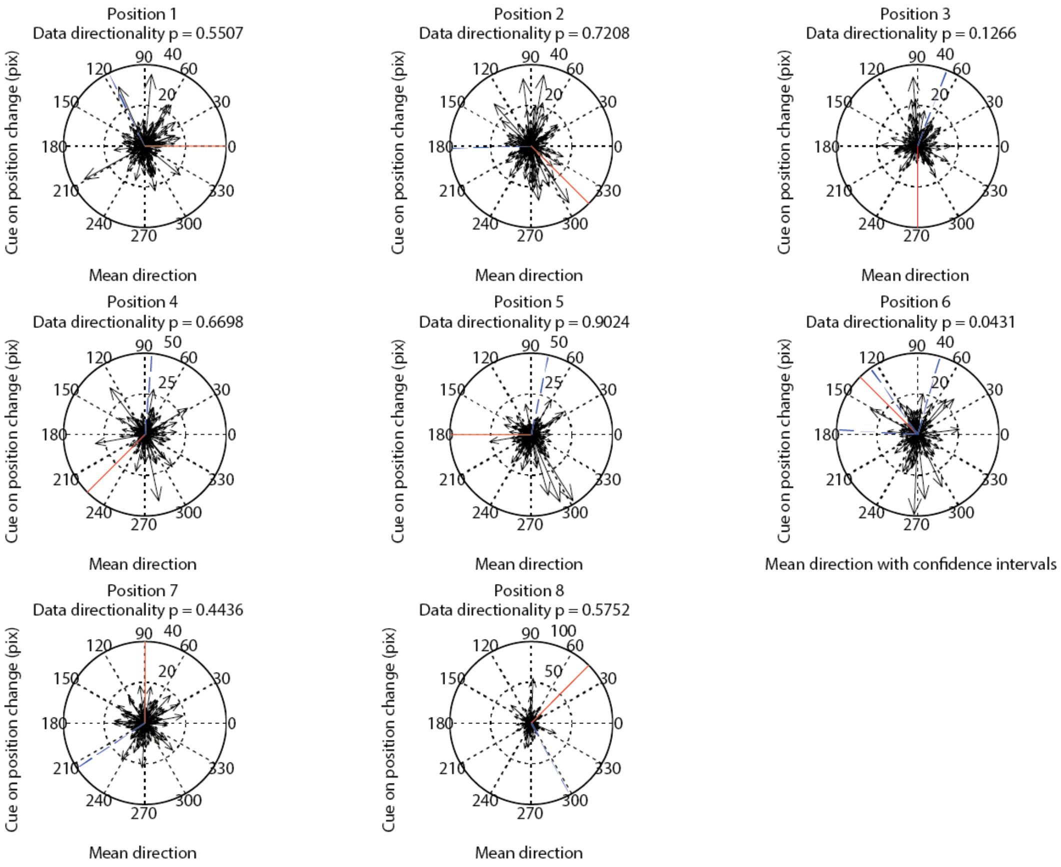

Exogenous

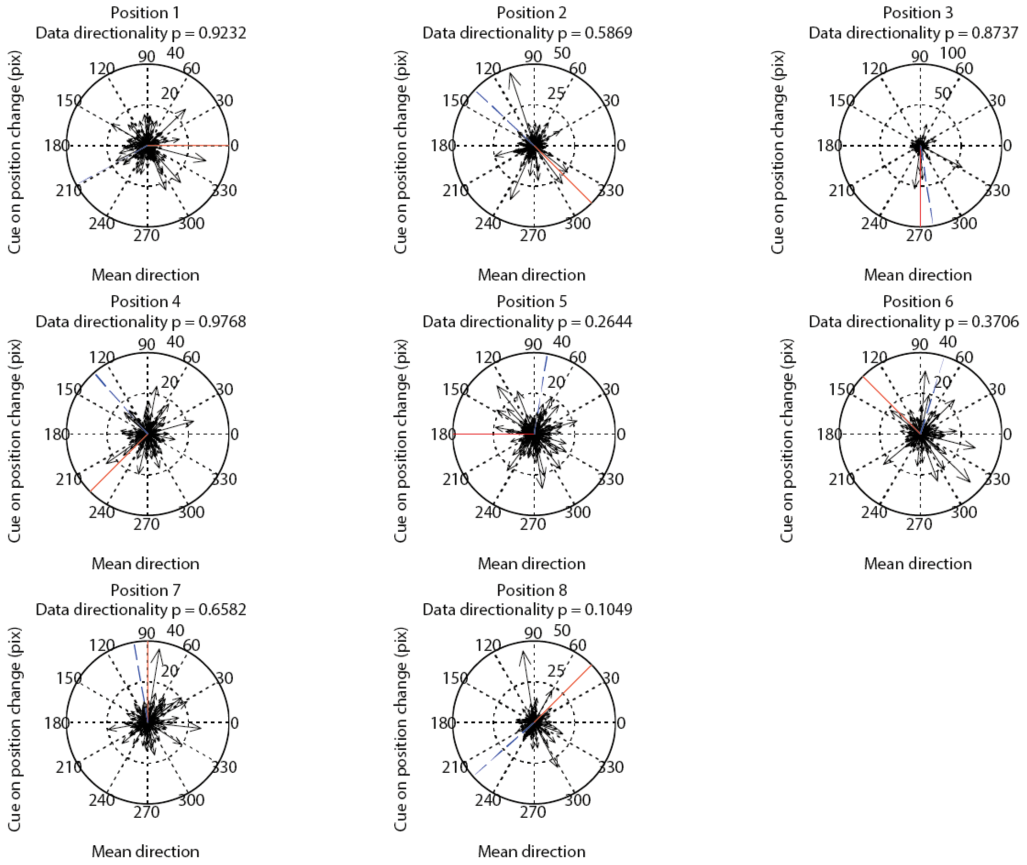

S 6: Directionality analysis of eye-movements. The data of all participants were pooled together. For every grating position in the attention conditions we calculated the median eye-position in the fixation period and in the cue-on period and using this calculated the change in position between fixation and cue period. We tested if there was any directionality in these eye position changes by using the Rayleigh test for non-uniformity from the CircStat toolbox for Matlab 53Top: Endogenous attention condition; Bottom: Exogenous attention condition. Every subplot of the figures corresponds to a grating position whose angle is indicated in red. Every black arrow corresponds to one trial. The length and direction of an arrow corresponds to the eye position change in pixels after cue onset. The circular mean of all angles of the position changes is indicated in blue. As can be seen the changes in eye position are very small (rarely exceeding 25 pixels) and are not directed towards the grating position, instead they are very dispersed. Accordingly p-values of the Rayleigh test were very high, only once falling below 0.05 (position 6 under exogenous attention).

S7 Analysis. Effect of Stimulus Onset Asynchrony (SOA). To rule out an effect of SOA we pooled the data of all subjects together and split trials in two halves according to SOA in the two attention conditions respectively. We then compared the longer half to the short half with a paired samples t-test. This yielded no significant effect of SOA on performance (endogenous: t (df = 1144) = -0.87, p = 0.39; exogenous: t (df = 1143) = -1.58, p = 0.11) and neither on confidence (endogenous: t (df = 1144) = 0.09, p = 0.93; exogenous: t (df = 1143) = 1.67, p = 0.1). An effect of stimulus onset asynchrony within conditions can be ruled out.

S8 Instruction sheet.

Experiment instructions:

You are taking part in a psychophysical experiment. We are trying to learn something about how visual stimuli are perceived and processed.

Throughout the experiment we will track your eye movements. So first we need to calibrate the eye-tracker. To do this there will be little targets appearing at different positions on the screen. Please look at every target and keep looking there until it disappears and the next one appears.

To ensure a quality of the eye data, please keep your head as still as possible in the headrest. Try to only remove it during the breaks you are offered (every quarter of the trials) If you accidentally move your head please tell the examiner because the Eye-Tracker will need to

be recalibrated. Additionally please try to only blink between the trials as this might also influence the quality of the data.

Please always look at the small black dot as long as it is presented on the screen.

During the experiment you will see gratings with or without cues. There will be an explanation trial, where you don’t have to fixate the fixation point and can look at the grating and the cues to get an impression how they look.

Your task will be to remember the orientation of a stimulus and then try to match the orientation of another equal stimulus to the orientation of the one you briefly saw before. You'll use the arrow keys on the keyboard to turn the second grating until it looks how you remember the first one. Don’t worry if in the beginning everything happens really fast and you have the feeling you are not seeing anything. That will get better with time.

At the end of every trial you will be asked to rate how confident you are in your performance in the orientation matching task. You shall do that using a scale, where green means high confidence and red means low confidence. The range between can be used to express different gradations.

Before the actual experiment starts there will be a training session that will get harder while you solve it and where you don’t have to make confidence ratings.

Do you have any questions?

Thanks in advance and have fun.
